# A Case of Nonfunctioning Pituitary Carcinoma That Responded to Temozolomide Treatment

**DOI:** 10.1155/2012/645914

**Published:** 2012-10-08

**Authors:** Haruko Morokuma, Takao Ando, Takuya Hayashida, Ichiro Horie, Naoko Inoshita, Fumi Murata, Ikuko Ueki, Kan Nakamura, Misa Imaizumi, Toshiro Usa, Atsushi Kawakami

**Affiliations:** ^1^First Department of Medicine, Nagasaki University Graduates School of Biomedical Sciences, 1-7-1 Sakamoto, Nagasaki 852-8501, Japan; ^2^Department of Pathology, Toranomon Hospital, 2-2-2 Toranomon, Minato-ku, Tokyo 105-8470, Japan

## Abstract

Pituitary carcinoma is a rare malignancy and is difficult to manage. Pituitary carcinomas commonly produce either PRL or ACTH, but some do not produce pituitary hormones. The alkylating reagent temozolomide (TMZ) was recently shown to be effective as a treatment for pituitary carcinoma. Most of the published reports of TMZ use in pituitary carcinoma cases were against hormone-producing carcinomas. Only a few patients with a nonfunctioning pituitary carcinoma treated with TMZ have been reported. Here we describe our treatment of a patient with nonfunctioning pituitary carcinoma and a background of multiple endocrine neoplasia type 1. The pituitary carcinoma was accompanied by meningeal dissemination with cerebral and L1 spinal bone metastasis. The patient received continuous dosing of TMZ along with external radiation, followed by standard dosing of TMZ. There was an apparent antitumor response seen in MRI. MGMT, an enzyme antagonized by TMZ, was negative in the tumor. The therapeutic efficacy of TMZ and dosing schedules of TMZ in pituitary carcinoma are discussed.

## 1. Introduction

Pituitary carcinomas are quite rare; they comprise only about 0.1% of pituitary tumors. A diagnosis of pituitary carcinoma is made clinically based on the presence of a pituitary tumor with metastasis, and not by microscopic findings of tumor invasion [1, 2]. It is also critical to exclude other cancer(s) that could explain the patient's clinical picture. Several carcinogenic events are believed to be involved in a developing pituitary carcinoma, since the diagnosis of a pituitary carcinoma is commonly made 5 to 10 years after the initial pituitary surgery [1, 3]. This concept is also supported by higher p53 expression and MIB-1 indices in the recurrent tumor tissues compared to the primary tumor tissues [4–6]. The prognosis for patients with pituitary carcinoma is on average only 2.6 years—when the carcinoma confined to the central nervous system. This is because pituitary carcinoma is highly aggressive and resistant to conventional treatments.

Temozolomide (TMZ) was recently shown to be an effective chemotherapeutic reagent for pituitary carcinoma treatment [1, 3, 7, 8]. TMZ is an oral alkylating reagent used in the treatment of refractory glioblastoma multiforme [9] and neuroendocrine tumors [10]. Lim et al. [11] reported the first case of pituitary carcinoma successfully treated with TMZ. The successful use of TMZ has been reproduced in several patients with pituitary carcinoma or refractory atypical pituitary adenoma [12–14]. However, it is also not known if the current protocol of TMZ treatment is ideal for treating pituitary carcinoma.

Here we report the case of a patient with nonfunctioning pituitary carcinoma successfully treated with TMZ because only few patients with a nonfunctioning pituitary carcinoma treated with TMZ have been reported. Remarkable tumor reduction and clinical improvement were obtained. Since the patient was treated with a nonstandard protocol with TMZ, we also discuss TMZ protocols.

## 2. Case Report

A 58-year-old male was admitted to our university hospital in January 2011 because of his general fatigue, weight loss, and occipital headache associated with hyponatremia and hypercalcemia. None of his family members suffered from endocrinological disorders. His past medical history was marked with urolithiasis at age 42. He had also had bilateral hemianopsia, and he had undergone transsphenoidal surgery against nonfunctioning pituitary adenoma in a local hospital at age 53 in 2006. After the surgery, his visual acuity and bilateral hemianopsia improved. However, his visual acuity decreased again one year after the pituitary surgery. The recurrent pituitary adenoma was then partially dissected, followed by external irradiation (50 Gy) in 2007. This treatment reduced the pituitary tumor, and thus the patient's visual acuity improved.

At admission to our hospital, the patient's physical examination was not remarkable. His visual acuity was not decreased and there were no defects in the visual field. Blood tests showed mild hyponatremia (132 mEq/L) and hypercalcemia (11.2 mg/dL). There was pituitary insufficiency (Table 1). The serum level of intact PTH was also high (252.4 pg/mL, reference range 10.3–65.9 pg/mL), and thus his hypercalcemia seemed to be due to primary hyperparathyroidism.

**Figure 1:**
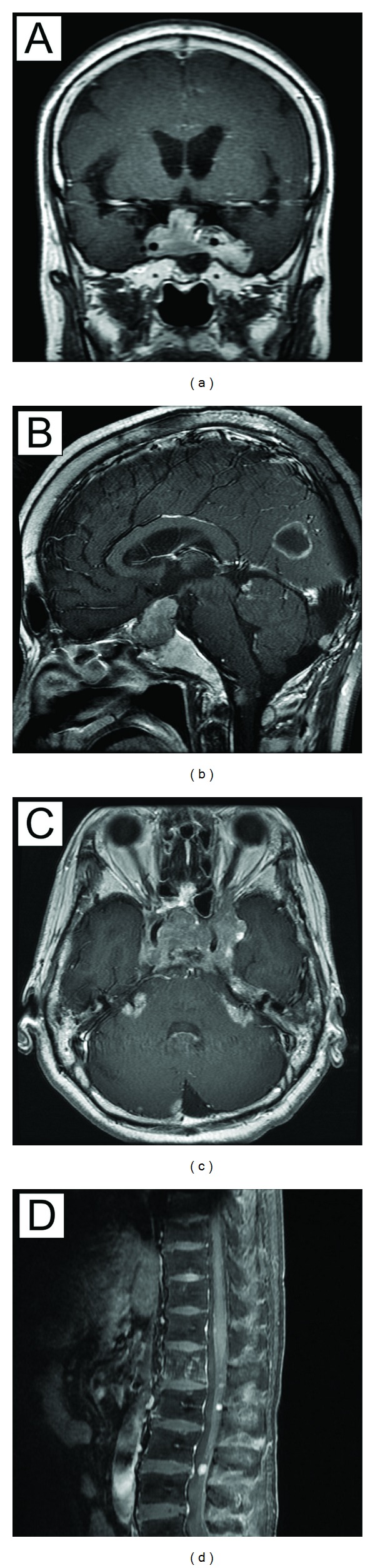
Pituitary carcinoma before the treatment. The pituitary tumor surrounds the bilateral cavernous sinus and protrudes close to the optic chiasm (detected by contrast-enhanced MRI). The tumor invades the left temporal lobe (a). Metastatic brain tumor in the occipital lobe shows ringlike enhancement (b). The pituitary tumor shows meningeal dissemination (c). Metastatic tumor in the L1 and diffuse meningeal dissemination (d).

Because of the patient's past history of pituitary tumor and present primary hyperparathyroidism, the patient was suspected to be a sporadic case of multiple endocrine neoplasia (MEN) type 1. To test this, a systemic survey was performed. First, a large pituitary tumor with a diffuse meningeal dissemination with multiple metastatic tumors in the brain and the L1 spine (Figure 1) was found. A lumbar punctuation to obtain cytology specimens was not performed because of the potential risk of the brain herniation. Secondly, a neck tumor with a marked accumulation of MIBI (not shown) was identified. Finally, multiple pancreatic tumors with increased vascularity, compatible with a neuroendocrine tumor (not shown) were identified. No other tumors were detected, and therefore the patient was clinically diagnosed as a sporadic case of MEN type 1 with a clinically nonfunctioning pituitary carcinoma, a parathyroid tumor, and pancreatic neuroendocrine tumors. Genomic analysis failed to identify mutation(s) in menin exons (data not shown).

After starting 20 mg of hydrocortisone followed by 25 *μ*g of levothyroxine, the patient recovered his appetite and his hyponatremia was corrected. However, severe orbital pain developed, and it was exacerbated upon light and sound. This additional symptom seemed to indicate rapid progression of the pituitary carcinoma.

**Figure 2:**
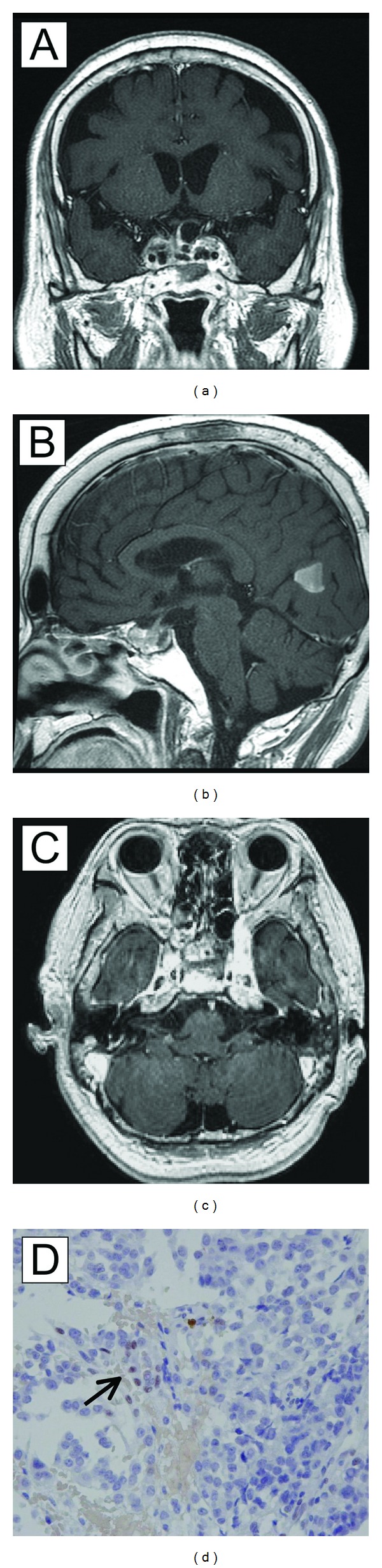
The pituitary carcinoma after 12 cycles of treatment. The pituitary tumor is decreased and limited mostly within the sella (contrast-enhanced MRI). The left temporal lobe is not invaded by the tumor (a). The metastatic brain tumor in the occipital lobe grew smaller, without ringlike enhancement (b). The stalk was then identifiable (a and b). The meningeal dissemination regressed markedly (c). MGMT expression was negative in the pituitary tumor cells. MGMT was positive in the endothelial cells (arrow) and served as the internal positive control. MGMT staining was performed as described in [15].

After informed consent including a potential risk to the additional radiation to the whole brain was obtained from the patient and his family, TMZ (75 mg/mm^2^ per day for 42 days) was initiated along with total brain irradiation (30 Gy) as well as monthly zoledronic acid (4 mg). Adverse effects were only minor, that is, hair loss in the irradiated area and mild bone marrow suppression. Thus, we continued the TMZ (192 mg/mm^2^ for 5 days every 28 days) starting four weeks after the initial 42-day treatment was completed. The pituitary carcinoma visibly declined (Figure 2) and the patient's complaints of periorbital pain and occipital pain also decreased. There was an apparent decrease of the meningeal dissemination in the spine (not shown). The patient has completed 20 cycles of TMZ (192 mg/mm^2^ for 5 days every 28 days) with continuous clinical efficacy.

We analyzed the pituitary tumor specimens obtained in the second pituitary surgery. There was no expression of GH, PRL, ACTH, TSH, LH, or FSH (not shown). The MIB-1 index and p53 positivity were increased by 7.6% and weakly 1.5%, respectively. We also examined MGMT (O^6^-methyl-guanine-DNA methyltransferase) expression because negative MGMT expression may be associated with a favorable response to TMZ. As expected, MGMT expression in the patient's pituitary tumor was negative (Figure 2).

## 3. Discussion

TMZ is a lipophilic alkylating reagent with a fair tissue distribution to the cerebrospinal fluid. Since TMZ causes only mild adverse effects, thus it can be used for a longer term of treatment than other cytotoxic reagents [8]. TMZ can induce apoptosis by methylating guanine to O^6^-methylguanines [7], and O^6^-methylguanine is then corrected to guanine by MGMT. In treatment with TMZ, MGMT specifically antagonizes TMZ although MGMT originally plays a protective role in carcinogenesis. Interestingly, when MGMT corrects O^6^methylguanine, MGMT is irreversibly inactivated, and thus TMZ also antagonizes MGMT. Therefore, it may be that TMZ treatment eventually depletes MGMT in tumor cells if the MGMT expression is low, or MGMT inactivation may be faster than MGMT synthesis [16]. It has been shown that TMZ was effective in malignant glioma with low mRNA and/or protein levels of MGMT [17].

Only a few cases of pituitary carcinoma successfully treated with TMZ have been reported until recently [11, 18, 19]. Some therapeutic responses of pituitary cancer to TMZ were reported very recently [12, 13, 20]. When these three studies are summarized, among the 21 cases described, 8 patients (38%) showed partial response, 5 (24%) showed stable disease, and 8 (38%) showed disease progression. Raverot et al. [8] recently reviewed the increasing number of cases of pituitary carcinoma and aggressive pituitary tumors treated with TMZ. They showed that low expression of MGMT seemed to better correlate with favorable therapeutic response and that intermediate to high MGMT expression seemed to better correlate with resistance to TMZ [8]. However, Raverot et al. pointed out the inconsistency of MGMT expression among researchers and, therefore, they suggested that positive MGMT expression might not be useful to exclude patients from receiving TMZ. Based on their findings, Raverot et al. contended that TMZ could be an important drug against aggressive pituitary tumors and pituitary carcinomas [8].

In a review of the literature by Dudziak et al. (2011), the majority of pituitary carcinomas produced either PRL (36%) or ACTH (30%) and the tumors producing other pituitary hormones were rare: GH 5%, TSH, GnRH (gonadotropin-releasing hormone), and LH (2% each). No hormonal secretion was seen in 23% [3]. However, only three patients with a nonfunctioning pituitary carcinoma treated with TMZ have been reported [7, 8]. It is not certain why most of the reported patients with a nonfunctioning pituitary carcinoma were not treated with TMZ. Since only successful cases tend to be reported, TMZ's antitumor effect might be poor in nonfunctioning pituitary carcinoma. Among patients with PRL- or ACTH-producing carcinomas, TMZ was shown to be effective in about three-fourths [7]. However, among three cases of nonfunctioning pituitary carcinoma [18, 20], TMZ was effective only in one patient [18]. Thus, the successful use of TMZ in our patient with nonfunctioning pituitary carcinoma would be important.

### 3.1. TMZ Protocols

The standard TMZ protocol in the treatment of pituitary cancer has been to administer TMZ 150–200 mg/mm^2^ for 5 days every 28 days for 12 cycles and then to withdraw the reagent. This protocol is the exact replication of the one used in the treatment of malignant glioma [7]. However, a nearly to 50% recurrence rate has been shown after withdrawal of TMZ in patients with an aggressive pituitary tumor or pituitary carcinoma [8]. Moreover, some patients with relapsed tumor showed resistance to TMZ in the second course [8, 18]. In contrast, good therapeutic response continued longer than 2 years in some patients with aggressive pituitary adenoma or carcinoma treated with TMZ without withdrawing the reagent [12, 13, 15, 21]. Therefore, it is currently not known how many TMZ cycles should be administered to a patient with pituitary carcinoma.

It is also not known if the current standard dosing schedule of TMZ mentioned above is ideal for treating pituitary carcinoma. For the treatment of progressive or recurrent glioblastoma multiforme, alternative dosing schedules of TMZ have been shown to be effective [7]. This included a metronomic protocol using continuous daily low-dose (50–75 mg/mm^2^) TMZ combined with radiation [22]. Metronomic dosing of TMZ has also been suggested to have additive antiangiogenic properties in vitro [23]. In addition, continuous dosing, including metronomic dosing, seems to be more effective in depleting MGMT because the regimen can deliver a higher cumulative dose over a prolonged period [16]. We therefore used metronomic dosing of TMZ along with radiation for our patient, because this treatment might enhance the therapeutic efficacy. After the metronomic dosing, the patient has been under the standard dosing for nearly two years. We will continue the standard dosing unless the carcinoma acquires resistance to TMZ, as reported previously [12, 13, 15, 21], or severe adverse effects develop. Nevertheless, it is not certain whether metronomic regimens are more effective than the standard protocol in the treatment of pituitary carcinoma.

In conclusion, we successfully treated a patient with nonfunctioning pituitary carcinoma with TMZ. We initially used a continuous dosing schedule to increase the cumulative dose of TMZ, and this was combined with external radiation. If the efficacy of the schedule is reproduced in more patients, a prospective study may be needed to compare the standard protocol with a continuous dosing protocol of TMZ.

## Figures and Tables

**Table 1:** The patient's pituitary hormones at admission.

Pituitary hormone	Result	Reference range
LH	0.10 mIU/mL	0.79–5.72
FSH	0.67 mIU/mL	2.00–8.30
Testosterone	0.05 ng/mL	2.01–7.50
GH	0.340 ng/mL	0.003–0.971
IGF-1	170 ng/mL	81–235
PRL	10.5 ng/mL	3.7–16.3
ACTH	33.6 pg/mL	7.0–56.0
Cortisol	4.1 *μ*g/dL	4.5–21.1
Free T3	2.36 pg/mL	2.1–4.1
Free T4	0.92 ng/dL	0.95–1.74
TSH	3.470 *μ*IU/mL	0.38–3.64
